# The Gut Microbiome in Early-Onset Colorectal Cancer: Distinct Signatures, Targeted Prevention and Therapeutic Strategies

**DOI:** 10.3390/jpm15110552

**Published:** 2025-11-12

**Authors:** Sara Lauricella, Francesco Brucchi, Roberto Cirocchi, Diletta Cassini, Marco Vitellaro

**Affiliations:** 1 Colorectal Surgery Unit, Fondazione IRCCS Istituto Nazionale Dei Tumori, 20133 Milan, Italy; 2 Hereditary Digestive Tract Tumors Unit, Fondazione IRCCS Istituto Nazionale Dei Tumori, Via Giacomo Venezian 1, 20133 Milan, Italy; 3 General Surgery Residency Program, University of Milan, 20122 Milan, Italy; francesco.brucchi@unimi.it; 4 Digestive and Emergency Surgery Unit, Santa Maria Hospital Trust, 05100 Terni, Italy; roberto.cirocchi@unipg.it; 5 Unit of General Surgery, Sesto San Giovanni Hospital, ASST Nord Milano, 20099 Sesto San Giovanni, Italy; diletta.cassini@asst-nordmilano.it

**Keywords:** early-onset colorectal cancer, gut microbiome, microbiota, dysbiosis, microbial signatures

## Abstract

**Background/Objectives:** The incidence of early-onset colorectal cancer (EOCRC) is rising worldwide, although its biological and clinical features remain incompletely understood. Emerging evidence implicates gut microbial dysbiosis as a key driver of EOCRC pathogenesis, acting through complex interactions with host genetics, mucosal immunity, and early-life exposures. This review synthesizes current evidence on EOCRC-specific microbial signatures, delineates host–microbiome interactions, and evaluates how these insights may inform precision prevention, early detection, and therapeutic strategies. **Methods:** A systematic literature search was conducted in PubMed, Scopus, and Web of Science up to August 2025, using combinations of “early-onset colorectal cancer,” “gut microbiome,” “dysbiosis,” and “host–microbiome interactions.” Both clinical and preclinical studies were included. Extracted data encompassed microbial composition, mechanistic insights, host-related factors, and microbiome-targeted interventions. Evidence was synthesized narratively to highlight consistent patterns, methodological limitations, and translational implications. **Results:** EOCRC is consistently associated with enrichment of pro-inflammatory and genotoxic taxa (e.g., *Fusobacterium nucleatum*, colibactin-producing *Escherichia coli*, enterotoxigenic *Bacteroides fragilis*) and depletion of short-chain fatty acid–producing commensals. Multi-omics analyses reveal distinct host–microbiome signatures influenced by germline predisposition, mucosal immunity, sex, and early-life exposures. However, substantial methodological heterogeneity persists. Collectively, these data point to candidate microbial biomarkers for early detection and support the rationale for microbiome-targeted preventive and adjunctive therapeutic approaches. **Conclusions:** EOCRC harbors unique microbial and host–environmental features that distinguish it from late-onset disease. Integrating host determinants with microbiome signatures provides a framework for precision prevention and tailored therapeutic strategies. Future priorities include harmonizing methodologies, validating microbial biomarkers in asymptomatic young adults, and rigorously testing microbiome-targeted interventions in clinical trials.

## 1. Introduction

Colorectal cancer (CRC) is the second most commonly diagnosed cancer and the second leading cause of cancer-related mortality in Europe, with over 520,000 new cases and 250,000 deaths reported across 40 countries in 2020 [1,2].

While the incidence of late-onset CRC (LOCRC, traditionally ≥ 50 years) has declined following the widespread adoption of screening programs, the incidence of early-onset CRC (EOCRC, <50 years) continues to rise, largely independent of hereditary cancer syndromes, highlighting an urgent and still poorly understood epidemiological shift [3,4].

Recent population-based studies report annual increases in EOCRC incidence ranging from 4 to 8% across several countries [5,6].

Although overall CRC-related mortality in younger adults remains relatively low, some countries are now observing unfavorable mortality trends within this age group [7].

Emerging evidence suggests that EOCRC is biologically distinct from LOCRC. Germline analyses in sporadic CRC have shown that patients under 50 more frequently carry pathogenic germline variants than those with LOCRC [8], suggesting that EOCRC may arise through distinct genetic and epigenetic mechanisms.

Indeed, early-onset tumors exhibit unique molecular features, including divergent somatic mutation profiles and distinct DNA methylation patterns, compared with their late-onset counterparts [5,9].

A range of lifestyle and environmental exposures—including diet, antibiotic use, obesity, physical inactivity, and other early-life influences—have been implicated in EOCRC pathogenesis. Increasing attention has turned to the gut microbiome as a potential mediator of the relationship between these exposures and CRC development. Epidemiologic data indicate that EOCRC-associated risk factors (e.g., Western-style diet, antibiotic use) can perturb the gut microbial ecosystem, supporting a “multi-hit” model in which chronic dysbiosis interacts with host metabolic, inflammatory, and immune pathways to promote early tumor initiation.

In this review, we explore how internal (host-related) and external (environmental) factors shape the gut microbiome and contribute to the rising incidence of EOCRC. We also discuss specific microbial signatures in EOCRC, with a focus on emerging microbiota-targeted strategies for prevention and precision therapy.

## 2. Methods

Relevant literature on the gut microbiome in early-onset colorectal cancer (EOCRC) was identified through PubMed/MEDLINE (National Library of Medicine, Bethesda, MD, USA) and Scopus (Elsevier, Amsterdam, The Netherlands) using the keywords “*early-onset colorectal cancer*,” “*gut microbiome*,” “*microbiota*,” “*dysbiosis*,” and “*microbial signatures*.” Additional studies were retrieved by screening the reference lists of selected articles. Only publications in English, available up to May 2025, were included. Priority was given to original research articles, meta-analyses, and authoritative reviews addressing microbial composition, functional pathways, host–microbiome interactions, and potential diagnostic or therapeutic implications in EOCRC.

Data collection, reference management, and screening were performed using EndNote X9.3.3 (Clarivate Analytics, Philadelphia, PA, USA) and Microsoft Excel 2021 (Microsoft Corp., Redmond, WA, USA).

For each eligible study, we documented the analytical methods employed, including shotgun metagenomics, metabolomics, MWAS, qPCR, and WGS, to capture differences in resolution and methodological scope. (Table 1).

## 3. Microbial Dysbiosis as a Driver of Colorectal Carcinogenesis

Dysbiosis, defined as an imbalance in the gut microbial ecosystem, has been increasingly recognized as a contributor to colorectal carcinogenesis via multiple, interrelated mechanisms. These include chronic mucosal inflammation, epithelial barrier disruption, immune modulation, genotoxin production, and alterations in microbial metabolites.

Metagenomic studies have consistently demonstrated that the gut microbiota of CRC patients significantly differs from that of healthy individuals, with enrichment of pro-inflammatory and genotoxic taxa such as *Fusobacterium nucleatum*, *Bacteroides fragilis*, and *Escherichia coli* strains harboring the *pks* pathogenicity island [19,20,21,22,23].

These bacteria promote tumorigenesis through different mechanisms. For example, *Fusobacterium nucleatum* expresses FadA adhesin, which activates β-catenin signaling and inhibits T-cell–mediated immunity, thereby facilitating tumor immune evasion [24]. *Escherichia coli* harboring the pks island produces colibactin, a genotoxin that induces DNA damage. Additionally, microbial metabolites such as hydrogen sulfide and secondary bile acids can create a carcinogenic niche in the colon [19,20,21,22,23].

In parallel, multiple studies have reported a depletion of beneficial commensal bacteria—such as *Faecalibacterium prausnitzii*, *Bifidobacterium*, *Ruminococcus*, and members of the *Lachnospiraceae* family—which produce short-chain fatty acids (SCFAs), crucial for maintaining epithelial integrity and immune homeostasis [25,26,27]. This recurring dysbiotic pattern, observed in fecal and mucosal samples from different populations, has been proposed as a potential microbial signature of CRC [10,11,12].

Beyond compositional changes, the gut microbiota also influences carcinogenesis through metabolic and functional pathways: microbial-derived peptides, toxins, and metabolites modulate tumor behavior by affecting processes such as apoptosis, cell proliferation, and immune surveillance [13].

These insights have led to increasing interest in microbiota-targeted interventions—such as dietary modulation, prebiotics, probiotics, and fecal microbiota transplantation (FMT)—as adjuncts to CRC prevention and therapy [13,14,28]. However, clinical implementation of these approaches remains limited by heterogeneity in study protocols, small sample sizes, and the inherent complexity of host–microbiome interactions.

## 4. Microbial Signatures in EOCRC

Emerging evidence indicates that EOCRC harbors distinct gut microbial and metabolic profiles that may contribute to its aggressive clinical behavior and divergent pathogenesis compared with LOCRC. Patients with EOCRC typically exhibit enrichment of pro-inflammatory and oncogenic taxa—including *Fusobacterium nucleatum*, enterotoxigenic *Bacteroides fragilis* (ETBF), colibactin-producing (*pks*^+^) *Escherichia coli*, and *Peptostreptococcus anaerobius*—together with depletion of anti-inflammatory, SCFA-producing commensals such as *Faecalibacterium prausnitzii*, members of the Lachnospiraceae family, and *Bifidobacterium* [10]. These microbial shifts promote carcinogenesis through genotoxin production, chronic mucosal inflammation, and modulation of the tumor immune microenvironment.

At the mechanistic level, *Fusobacterium nucleatum* activates Wnt/β-catenin signaling via the adhesin FadA and suppresses anti-tumor immunity through Fap2-mediated inhibition of NK and T cells [11,12]. ETBF secretes the BFT toxin, which stimulates STAT3 and Th17 inflammatory cascades, fostering a pro-tumorigenic milieu [13].

*pks*^+^ *E. coli* produces colibactin, a genotoxin that induces DNA crosslinks and generates the mutational signature SBS88, recently identified in EOCRC tumors [14]. *Peptostreptococcus anaerobius* enhances tumorigenesis by inducing reactive oxygen species and activating PI3K–Akt signaling [28]. Collectively, these mechanisms underscore biologically plausible microbial drivers, yet most EOCRC studies remain associative, highlighting the need for longitudinal, interventional, and multi-omic approaches to establish causality.

Western-style diets may further reinforce this dysbiosis by favoring sulfur-metabolizing and pro-inflammatory taxa that generate hydrogen sulfide, a genotoxin implicated in DNA damage and tumorigenesis [17,29]. Multi-omics studies corroborate these age-dependent differences. In a cohort of 460 CRC patients (including 167 EOCRC), Adnan et al. reported stronger tumor–microbiome interactions and distinct enrichments in EOCRC compared with LOCRC [17]. Similarly, Kong et al. identified EOCRC-specific microbial and metabolic phenotypes—including enrichment of *Flavonifractor plautii* and alterations in tryptophan, choline, and bile acid metabolism—not observed in older patients [30]. A landmark genomic analysis of ~1000 CRC cases across 11 countries further revealed colibactin-associated mutational signatures (SBS88 and ID18) that were 3.3-fold more frequent in CRC < 40 years than in >70 years, accounting for ~15% of APC mutations in the younger cohort [18].

Conversely, some studies suggest overlap across age groups: a cross-sectional analysis found enrichment of canonical CRC-associated bacteria (*Clostridium symbiosum*, *Peptostreptococcus stomatis*, *Parvimonas micra*, *Hungatella hathewayi*, *Fusobacterium nucleatum*, *Bacteroides fragilis*) in both EOCRC and LOCRC, with conserved virulence factors such as FadA and BFT [16]. Taken together, these findings suggest that while a core set of oncobionts persists across colorectal tumors, broader microbial networks and host interactions in younger patients may differ substantially, potentially accelerating tumorigenesis.

## 5. Host-Microbiome Interactions in Young Patients

The increasing incidence of EOCRC cannot be fully explained by germline predisposition alone. Recent multi-omics studies have identified distinct microbial and host-transcriptomic profiles in EOCRC, highlighting the role of aberrant microbiome-host crosstalk established early in life. This interaction may foster a mucosal microenvironment permissive to tumor initiation [15,31].

**Figure 1:**
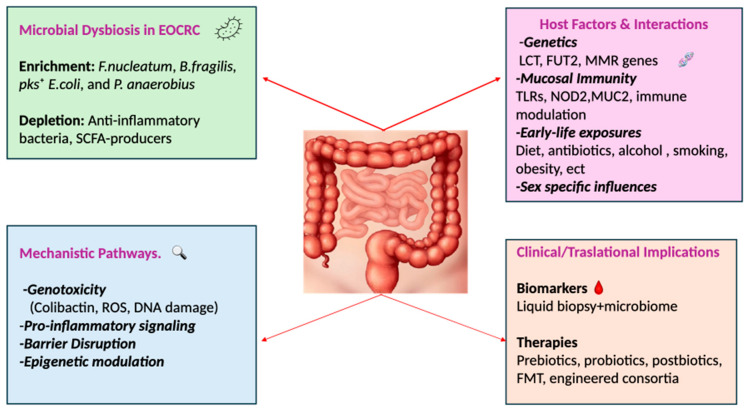
EOCRC-associated microbiome and host interactions.

We explore how host-related factors—including genetics, mucosal immunity, sex-based differences, and early-life exposures—shape gut microbial composition and function in ways that may modulate individual susceptibility to EOCRC (Figure 1).

### 5.1. Host Genetics

Although host genetic variation exerts only a modest influence on gut microbiome composition, even subtle variants may shape mucosal immunity and host-microbe interactions, thereby contributing to EOCRC risk. A large genome-wide association study of >18,000 individuals showed that common genetic variants explain < 5% of variation in gut microbiome composition. Notably, polymorphisms in genes involved in diet-microbe interactions and mucosal biology, such as the lactase (LCT) gene and the FUT2/ABO secretor locus, have been linked to shifts in specific taxa, including *Bifidobacterium*, in an allele- and diet-dependent manner [32]. Variants in mucin genes (e.g., MUC2), innate immune receptors (e.g., TLRs, NOD2), and host antimicrobial peptides may further shape microbial colonization at the mucosal interface.

Importantly, EOCRC patients more frequently harbor pathogenic germline variants compared with LOCRC, suggesting that inherited susceptibility may interact with microbial exposures to accelerate tumorigenesis. For example, mismatch repair deficiency, as in Lynch syndrome, is associated with enrichment of pro-inflammatory taxa. Similarly, alterations in genes regulating barrier integrity or immune surveillance may amplify the oncogenic effects of pathobionts such as colibactin-producing *Escherichia coli* or *Fusobacterium nucleatum*.

Thus, while environmental and lifestyle factors remain dominant drivers of the EOCRC microbiome, host genetic variation—particularly when affecting mucosal immunity or barrier function—may modulate individual susceptibility to dysbiosis-driven carcinogenesis [33]. Future studies integrating host genomics with metagenomics and metabolomics will be critical to disentangle these complex interactions and to identify high-risk subgroups amenable to precision prevention strategies.

### 5.2. Mucosal Immune Control

The gut mucosal immune system represents a highly dynamic interface through which the host exerts selective control over microbial communities. Secretory IgA, host antimicrobial peptides (e.g., defensins), and host-derived microRNAs contribute to microbial homeostasis by suppressing pathobionts and supporting commensal taxa, thereby shaping both the composition and functional output of the gut microbiota [34].

In EOCRC, host-microbiome immune interactions appear particularly pronounced. Young-onset tumors are more frequently colonized by *Fusobacterium nucleatum* and exhibit stronger Th1/Th17 immune responses compared with LOCRC [35], suggesting that younger hosts mount a distinct mucosal immune profile—potentially driven by a higher microbial immunogenic load—that may modulate tumor behavior. More broadly, immune factors such as baseline immune tolerance, checkpoint regulation, inflammation tone, and mucosal barrier integrity collectively determine whether microbial dysbiosis is contained or permitted to contribute to carcinogenesis. For instance, compromised epithelial barrier or defective regulatory T-cell activity in younger individuals may facilitate microbial translocation and chronic mucosal inflammation, thereby accelerating tumor initiation [36].

Younger patients may be particularly vulnerable to dysbiosis-driven immune modulation. Compared with older adults, individuals under 50 display an expanded pool of naïve immune cells and heightened innate responsiveness which—while advantageous for pathogen defense—can lead to exaggerated inflammatory and regulatory responses to microbial products, amplifying mucosal damage [37,38,39]. Limited cumulative antigen exposure and reduced “trained” immunity further magnify the impact of novel microbial toxins, while greater genetic and transcriptional variability in immune pathways may heighten interindividual susceptibility [38]. Collectively, these features suggest that identical microbial insults, such as colibactin exposure or SCFA depletion, may exert disproportionately stronger oncogenic effects in younger hosts, thereby shaping the distinct biology of EOCRC.

### 5.3. Sex

In addition to genetic and immunological factors, host sex influences gut microbial ecology. In the general population, males and females display consistent differences in microbiota composition, partly shaped by sex hormones, with downstream effects on immunity, metabolism, and mucosal barrier function [35,40,41]. Notably, sex-related di-vergence has also been reported in CRC, including enrichment of *Bacteroides* spp. in males and *Bifidobacterium* in females, in line with hormone-dependent modulation of mucosal immunity and metabolic tone [42,43].

By contrast, direct evidence for sex-specific microbial signatures in EOCRC remains scarce. Several studies have characterized the EOCRC microbiome, reporting enrichment of *Fusobacterium* and *Escherichia*–*Shigella* alongside depletion of butyrate producers, but few have performed sex-stratified analyses or reported reproducible sex-specific patterns [44,45]. One study noted that microbial richness and abundance may vary by host factors including sex, but detailed profiles were not provided and validation in EOCRC is lacking [46]. Recent reviews emphasize this gap, underscoring the absence of large-scale, sex-stratified EOCRC cohorts and the urgent need for harmonized, adequately powered studies to determine whether sex modifies EOCRC microbial signatures [47,48].

Taken together, while sex is a plausible modifier of the CRC microbiome, robust and reproducible sex-specific microbial patterns in EOCRC have not yet been demonstrated. Addressing this gap will be essential to refine risk stratification and move toward truly personalized prevention and therapeutic strategies.

### 5.4. Early Life and Lifestyle Exposures

Beyond intrinsic factors, extrinsic exposures during early life and adulthood play a critical role in shaping the gut microbiome and modulating CRC risk. Dietary patterns—particularly Western-style diets rich in fat and poor in fiber –can induce gut dysbiosis characterized by enrichment of sulfur-metabolizing and pro-inflammatory bacteria [49,50].

Prospective data support this diet-microbiome-cancer axis: In the Nurses’ Health Study II, women under 50 with high adherence to a Western dietary pattern had a significantly increased risk of high-risk adenomas (OR 1.67; 95% CI 1.18–2.37), particularly in the distal colon and rectum. Conversely, adherence to prudent or fiber-rich dietary patterns (e.g., DASH or alternative Mediterranean diets) was associated with a 30–45% reduction in adenoma risk [51]. These findings highlight the potential of modifiable dietary exposures, especially in early adulthood, to influence carcinogenesis, suggesting that targeted nutritional interventions could represent a viable preventive strategy in younger adults.

Similarly, antibiotic exposure during infancy or early adulthood can affect normal gut microbial colonization, altering microbial diversity, reducing anaerobic commensals, and promoting a pro-inflammatory milieu [52]. Epidemiological data suggest that early-life antibiotic use is associated with increased risk of both EOCRC and LOCRC, potentially through long-term microbial perturbations that favor oncogenic taxa and suppress beneficial commensals [53].

Other lifestyle-related exposures commonly enriched in EOCRC_ such as sedentary behavior, excessive alcohol intake, smoking, and obesity_ have also been shown to shape gut microbiota [4,40,54]. These factors are independently associated with reduced microbial diversity and increased abundance of pro-inflammatory taxa. Obesity, in particular, promotes chronic inflammation and insulin resistance while reshaping the gut microbiota in ways that may favor neoplastic progression [4,54].

When these exposures occur early in life, they may induce durable shifts in the gut ecosystem that persist into adulthood, setting the mucosal environment for tumor development. This cumulative impact supports a “multi-hit” model, in which sustained dysbiosis, coupled with dietary, metabolic, and inflammatory stressors, accelerates carcinogenesis. Understanding this dynamic may guide future preventive strategies, including precision nutrition, microbial modulation, and microbiota-derived biomarker profiling for early-risk stratification.

## 6. Gut Microbiome as a Predictor of Therapeutic Response in CRC: Insights from EOCRC

Emerging evidence suggests that gut microbiota composition profoundly modulates therapeutic efficacy in CRC. Dysbiosis may influence drug metabolism, reshape antitumor immune responses, and alter the abundance of metabolites that impact tumor growth [55,56].

In particular, increased microbial diversity and enrichment of SCFA-producing taxa (such as *Faecalibacterium prausnitzii*, *Roseburia*, and *Blautia*) have been consistently associated with improved responses to immune checkpoint inhibitors (ICIs) and neoadjuvant chemoradiotherapy (nCRT) in rectal cancer [56,57,58]. Patients harboring richer commensal communities often display a metabolically favorable tumor microenvironment, characterized by elevated levels of butyrate and acetate, enhanced mucosal integrity, and reduced baseline inflammation.

Conversely, reduced microbial richness and increased abundance of pathogenic or oral-origin anaerobes—such as *Fusobacterium nucleatum*, *Peptostreptococcus*, *Escherichia coli*, and *Parvimonas micra*—have been linked to chemoresistance, suboptimal pathological responses, and poorer prognosis [59,60,61]. For instance, *Fusobacterium nucleatum* has been shown to impair the efficacy of 5-fluorouracil–based therapies and is associated with a higher risk of recurrence [62]. Similarly, non-responders to ICIs often harbor microbial signatures enriched in *Micrococcaceae* or *Enterobacteriaceae*, along with a depletion of protective taxa such as *Akkermansia muciniphila* [58].

Notably, recent studies suggest that EOCRC may represent a microbiologically distinct subtype with specific microbial patterns associated with treatment response. In a study by White et al., young-onset rectal cancer patients (n = 37) exhibited a unique intratumoral microbiota compared to older individuals (n = 71), characterized by increased abundance of *Escherichia coli*, *Parvimonas micra*, and *Clostridium symbiosum*, alongside a relative depletion of taxa more prevalent in late-onset cases (e.g., *Clostridium perfringens*) [59]. Within the EOCRC cohort, nCRT responders harbored higher levels of SCFA-producing and anti-inflammatory bacteria_ *Eggerthella lenta*, *Bacteroides dorei*, *Ruminococcus bromii*, whereas non-responders exhibited enrichment of oral-origin anaerobes.

Collectively, these findings underscore the emerging role of tumor-associated microbial signatures as predictive biomarkers, with the potential to stratify patients and tailor therapeutic interventions based on microbial composition.

## 7. Microbiome-Based Therapeutic Strategies in EOCRC

Growing evidence implicating the gut microbiome in colorectal carcinogenesis has spurred the development of microbiota-directed interventions as complementary strategies for prevention and therapy. These approaches aim to restore eubiosis, strengthen mucosal barrier integrity, and redirect microbial metabolism toward anti-cancer functions. Several modalities are currently under investigation.

Probiotics, particularly selected strains of Lactobacillus and Bifidobacterium, have shown promise in modulating inflammation, reinforcing epithelial barrier function, and suppressing oncogenic taxa. Certain Lactobacillus strains can induce tumor cell apoptosis and inhibit Wnt/β-catenin and NF-κB pathways, reducing polyp burden in animal models [63]. *Bifidobacterium* species have also been reported to enhance antitumor immunity and improve the efficacy of checkpoint blockade in preclinical studies [63,64].

Early-phase trials suggest that probiotics may mitigate chemotherapy-induced diarrhea and other toxicities, although benefits appear strain and dose-dependent. Large randomized trials remain necessary to define optimal regimens and validate efficacy in CRC prevention or as adjunct therapy [65,66]. Overall, the safety profile of probiotics is favorable, and *Lactobacillus* and *Bifidobacterium* remain key genera of interest, being associated with increased fecal butyrate and reduced pro-carcinogenic metabolites [63].

Prebiotics, such as fermentable fibers and oligosaccharides, selectively promote the growth of beneficial microbes. High-fiber or Mediterranean-style diets, rich in whole grains, fruits, and vegetables, are associated with greater microbial diversity, enrichment of SCFA-producing taxa, and reduced inflammation [19,67]. Interventions with resistant starch and inulin have been shown to increase *Faecalibacterium* and *Bifidobacterium* while suppressing colibactin-producing *Escherichia coli* [67]. Precision fiber formulations, such as butyrylated high-amylose maize starch (HAMSB), represent next-generation chemopreventive strategies by delivering butyrate directly to the colon [68].

Postbiotics, bioactive metabolites derived from non-viable bacteria, are emerging as a safer alternative to live probiotics [69]. Early studies suggest they may recapitulate many probiotic benefits—immune modulation, barrier reinforcement—while avoiding infection risks in immunocompromised patients [70,71]. Although clinical data remain limited, postbiotics represent a promising frontier for CRC prevention.

Fecal microbiota transplantation (FMT) has proven highly effective in Clostridioides difficile infection (>90% cure rates) [72]. In CRC, FMT is still experimental, aimed at restoring eubiosis, mitigating chemotherapy-related gut toxicity, and enhancing antitumor immunity [73]. Preclinical models show that FMT from healthy donors can inhibit tumor growth, modulate cytokine signaling, and improve immunotherapy responses [74,75,76]. However, its oncological application remains non-standard, with unresolved questions regarding safety, timing, and durability of effects.

Overall, microbiome-directed interventions in CRC are promising but remain preliminary. Randomized trials of probiotics and synbiotics—mainly *Lactobacillus* and *Bifidobacterium*—have improved barrier integrity and inflammatory markers, but consistent oncological benefits are lacking [77]. Prebiotics such as resistant starch and inulin show potential to restore SCFA production, though findings are heterogeneous [78]. FMT, while effective in *Clostridioides difficile* infection, has produced variable results in oncology, and EOCRC-specific data are absent [79,80]. Systematic reviews highlight heterogeneity in study design, dosing, and endpoints, underscoring the need for harmonized, adequately powered clinical trials.

These limitations have accelerated exploration of next-generation therapeutics, including rationally designed bacterial consortia, engineered commensals, and bacteriophage-based approaches, offering reproducible means of restoring microbial balance [81,82,83]. While still experimental, such strategies may be particularly relevant in EOCRC, where conventional interventions have yielded inconsistent results.

Taken together, microbiome-based therapies represent a rapidly advancing frontier. Future biomarker-driven trials—for example, stratifying by *pks^+^ Escherichia coli* carriage or SCFA depletion—will be crucial translation into clinical practice. Ultimately, integration of microbiome-targeted strategies with conventional treatments may enhance the precision and effectiveness of EOCRC management.

## 8. Microbiome-Based Implications for Diagnosis, Prognosis, and Future Directions

The identification of EOCRC-specific microbial signatures offers compelling opportunities for non-invasive diagnostic, prognostic, and therapeutic applications. Metagenomic stool profiling could substantially enhance current screening strategies, particularly in younger adults not yet eligible for colonoscopy. Composite microbial patterns—such as enrichment of *Fusobacterium nucleatum* alongside depletion of butyrate-producing taxa—are emerging as early-warning markers to prompt timely colonoscopic evaluation.

Several studies demonstrate that microbiota-based classifiers can distinguish CRC or advanced adenomas from healthy controls with promising sensitivity and specificity [84,85,86]. Validating these classifiers in asymptomatic, average-risk individuals under 50 represents a critical next step toward establishing their clinical utility in EOCRC screening.

Beyond diagnosis, the microbiome also provides prognostic insights. High intratumoral loads of *Fusobacterium nucleatum* have been consistently associated with worse survival, independent of tumor stage and molecular subtype [87,88]. If similarly enriched in EOCRC, such taxa could serve as prognostic biomarkers and guide intensified therapeutic strategies. In parallel, gut microbial composition has been implicated in shaping responses to immunotherapy and neoadjuvant regimens, suggesting that tumor or fecal profiling might help predict which EOCRC patients benefit most from microbiome-targeted interventions. Importantly, predictors established in late-onset cohorts may not be directly generalizable, underscoring the need for age-specific validation.

Integrated multi-omic analyses further demonstrate that combining gut microbial signatures (e.g., *F. nucleatum* enrichment, *Firmicutes* depletion) with serum metabolomic and liquid biopsy markers (ctDNA, microRNAs, exosomal RNAs) can achieve high diagnostic accuracy, with AUC values exceeding 0.9 in discovery cohorts [89]. Systematic reviews confirm that microbiome-informed classifiers improve performance when added to conventional tools such as fecal occult blood testing or multitarget stool DNA assays [90,91]. Prognostic applications are also emerging, integrating microbial features with liquid biopsy components to refine recurrence risk stratification and surveillance strategies [92,93,94].

Despite these encouraging advances, translation remains hampered by substantial heterogeneity in study design, biospecimen handling, and analytical pipelines. Most EOCRC studies are based on small, single-center cohorts, and data from underrepresented populations are limited. Earlier work relied heavily on 16S rRNA sequencing, which permits taxonomic profiling but lacks functional resolution, whereas more recent approaches employ shotgun metagenomics, metatranscriptomics, and metabolomics, enabling deeper mechanistic insight. Geographical and population biases further constrain interpretation: most studies have been conducted in high-income countries, while cross-cohort comparisons reveal profound ethnic and regional variation in microbial profiles [95,96,97]. Substantial interindividual variability—shaped by host genetics, diet, lifestyle, and antibiotic exposure—adds further complexity, complicating the identification of reproducible microbial signatures. Finally, the lack of standardized protocols for sample collection, together with the high costs of sequencing and analysis, continues to hinder reproducibility and clinical translation.

Addressing these challenges will require large-scale, multicenter, prospective studies with harmonized methodologies to disentangle disease-specific microbial features from background variability and to validate EOCRC-specific microbial biomarkers across diverse populations.

## 9. Conclusions

Microbiome-informed strategies hold transformative potential to reduce the burden of EOCRC. Standardized, multidisciplinary research approaches will be essential to distinguish causal microbial drivers from bystanders and to clarify underlying mechanisms. Ultimately, decoding and modulating the EOCRC-associated microbiome may open novel avenues for prevention, early detection, and personalized therapy in young adults.

This review contributes to the existing literature by synthesizing evidence across epidemiologic, mechanistic, and translational studies to highlight EOCRC-specific microbial signatures, their interaction with host factors, and their potential clinical implications. In doing so, it emphasizes that EOCRC is not merely an earlier manifestation of conventional CRC but a biologically distinct entity. By integrating mechanistic insights with emerging diagnostic and therapeutic strategies, this work provides a framework for future research and clinical translation aimed at precision prevention and tailored interventions in younger adults.

## Figures and Tables

**Table 1:** Summary of major EOCRC microbiome investigations and their methodological limitations.

Authors	Population	Methods	Key Findings	Limitations
Yu et al., 2017 [10]	74 CRC + 54 controls, validation in Denmark, France, Austria	Fecal metagenomics, MWAS, qPCR validation	Identified CRC-associated taxa (*Fusobacterium nucleatum*, *Parvimonas micra*, *Solobacterium moorei*); developed and validated non-invasive biomarkers with high diagnostic accuracy (AUC up to 0.84)	Limited sample sizes in validation cohorts; potential geographic bias
Rubinstein et al., 2013 [11]	Human CRC tissues, cell lines	Functional/mechanistic assays on FadA adhesin	Fusobacterium *nucleatum* FadA binds E-cadherin, activates β-catenin signaling, promotes CRC cell growth; FadA upregulated in adenomas and carcinomas	Mainly mechanistic, not large-scale clinical validation
Gur et al., 2015 [12]	Human CRC tissues, cell culture, immune assays	TIGIT–Fap2 interaction analysis	Fusobacterium nucleatum Fap2 protein inhibits NK and T-cell activity via TIGIT, enabling immune evasion	Focused on immune modulation; lacks epidemiological cohort
Wu et al., 2009 [13]	Mouse model (Min mice), ETBF vs. NTBF colonization	Colonization + immunological assays	ETBF induces colitis, Stat3/Th17 activation, and tumorigenesis; IL-17/IL-23 blockade prevents tumor formation	Preclinical study; limited human data
Pleguezuelos-Manzano et al., 2020 [14]	Human intestinal organoids; 5876 human cancer genomes	Organoid exposure to *pks*^+^ *Escherichia coli*, WGS, and mutational signature analysis.	Identified distinct mutational signature of colibactin in CRC; colibactin linked to APC driver mutations	Organoid model may not capture full in vivo complexity; prevalence in general population uncertain
Kong et al., 2023 [15]	114 EOCRC, 130 LOCRC, 197 controls; independent validation cohort	Multi-omics: metagenomics + metabolomics	EOCRC associated with Flavonifractor plautii, altered tryptophan/bile acid/choline metabolism; predictive multi-omics classifier performed well	Single-country cohorts; dietary/lifestyle confounders
Qin et al., 2024 [16]	Large yCRC and oCRC metagenomes from 2 independent cohorts (China)	Shotgun metagenomic sequencing	Consistent CRC microbial signatures (e.g., *Fusobacterium nucleatum*, *Bacteroides fragilis*) across young- and old-onset patients; microbiome-based models equally accurate across age groups	Mostly Chinese cohorts: functional validation limited
Adnan et al., 2024 [17]	701 CRC vs. 693 controls (fecal metagenomes, CMGData) + 85 tumor microbiomes (TCGA)	Bioinformatics, fecal and tumor microbiome, host transcriptomics	Age-specific microbial differences; stronger host–microbe interactions in EOCRC tumors	Secondary data analysis; heterogeneous datasets
Díaz-Gay et al., 2025 [18]	981 CRC genomes from 11 countries	WGS, mutational signature analysis	Geographic and age-related variation in mutational processes; SBS88/ID18 (colibactin) enriched in EOCRC; ~25% APC indels linked to colibactin	Correlation with microbiome exposure inferred, not directly measured

MWAS: Metagenome-Wide Association Study; qPCR: quantitative Polymerase Chain Reaction; WGS: Whole-Genome Sequencing.

## Data Availability

No new data were created or analyzed in this study. Data sharing is not applicable to this article.

## Abbreviations

CRC	Colorectal Cancer
EOCRC	Early-Onset Colorectal Cancer
LOCRC	Late-Onset Colorectal Cancer
SCFA	Short-Chain Fatty Acids
MWAS	Metagenome-Wide Association Study
qPCR	quantitative Polymerase Chain Reaction
WGS	Whole-Genome Sequencing
nCRT	Neoadjuvant Chemoradiotherapy
ICI	Immune Checkpoint Inhibitor
FMT	Fecal Microbiota Transplantation
HAMSB	Butyrylated High-Amylose Maize Starch
LCT	Lactase
